# Small molecule restoration of wildtype structure and function of mutant p53 using a novel zinc-metallochaperone based mechanism

**DOI:** 10.18632/oncotarget.2432

**Published:** 2014-09-03

**Authors:** Xin Yu, Adam R. Blanden, Sumana Narayanan, Lalithapriya Jayakumar, David Lubin, David Augeri, S. David Kimball, Stewart N. Loh, Darren R. Carpizo

**Affiliations:** ^1^ Rutgers Cancer Institute of New Jersey, New Jersey; ^2^ Department of Surgery, Rutgers Robert Wood Johnson Medical School, New Brunswick, New Jersey; ^3^ Department of Biochemistry and Molecular Biology, SUNY Upstate Medical University, Syracuse, New York; ^4^ Department of Medicinal Chemistry, Rutgers Ernest Mario School of Pharmacy, Piscataway, New Jersey

**Keywords:** mutant p53 reactivation, zinc-metallochaperone, mutant p53 targeted drug, thiosemicarbazone, reactive oxygen species (ROS)

## Abstract

NSC319726 (ZMC1) is a small molecule that reactivates mutant p53 by restoration of WT structure/function to the most common p53 missense mutant (p53-R175H). We investigated the mechanism by which ZMC1 reactivates p53-R175H and provide evidence that ZMC1: 1) restores WT structure by functioning as a zinc-metallochaperone, providing an optimal concentration of zinc to facilitate proper folding; and 2) increases cellular reactive oxygen species that transactivate the newly conformed p53-R175H (via post-translational modifications), inducing an apoptotic program. We not only demonstrate that this zinc metallochaperone function is possessed by other zinc-binding small molecules, but that it can reactivate other p53 mutants with impaired zinc binding. This represents a novel mechanism for an anti-cancer drug and a new pathway to drug mutant p53.

Significance: We have elucidated a novel mechanism to restore wild-type structure/function to mutant p53 using small molecules functioning as zinc-metallochaperones. The pharmacologic delivery of a metal ion to restore proper folding of a mutant protein is unique to medicinal chemistry and represents a new pathway to drug mutant p53.

## INTRODUCTION

*TP53* is the most commonly mutated gene in human cancer for which no effective targeted anti-cancer drug exists [1]. The majority of p53 mutations (>70%) are missense, and generate a defective protein that is found at high levels in cells due to the impairment of Mdm2 mediated negative feedback [2-4]. Restoration of p53 function in mouse tumor models has been shown to be highly therapeutic, thus reactivating mutant p53 pharmacologically has been a highly sought after goal in anti-cancer drug development [5-7].

We recently identified NSC319726 (hereafter zinc metallochaperone-1, or ZMC1) as a mutant p53 reactivator and lead compound for mutant p53 targeted drug development [8]. We observed that ZMC1 displayed allele specific effects in that it reactivated the most common missense mutant, p53-R175H, but not the R248 or R273 mutants. ZMC1 selectively killed p53-R175H cancer cells through the restoration of wild-type (WT) structure/function of the p53-R175H and initiation of a p53-mediated apoptotic program. These results we also observed *in vivo* where ZMC1 inhibited xenograft tumor growth in a p53-R175H dependent manner. ZMC1 belongs to the family of thiosemicarbazone metal ion chelators with affinity for cations such as Fe^2+^, Zn^2+^, Cu^2+^ and Mn^2+^ [9]. The mechanism of ZMC1 mediated p53-R175H reactivation is currently unknown. Initially two properties of the compound were identified that are important for anti-tumor activity: zinc binding and redox changes [9].

Structural studies of WT p53 have shown that p53 requires a single zinc ion (coordinated by four amino acids C176, H179 on the L2 loop, and C238, and C242 on the L3 loop) for proper folding [10, 11]. There is now ample biochemical and cellular evidence that manipulating zinc concentrations can change the structure/function of WT p53, indicating that the p53 structure is malleable [11-13]. A model of zinc-dependent folding and misfolding for p53 was proposed by Loh and colleagues, in which p53 is properly folded only when one molecule of zinc binds to the DNA binding domain (DBD; residues 94-312) [11, 14, 15]. A deficit of zinc results in loss of DNA binding specificity, a surplus leads to misfolding and aggregation. DBD misfolding is due to binding of zinc to one or more non-native sites in p53 in conditions where zinc is in excess. DBD contains 10 Cys and 9 His residues that can potentially bind zinc.

In this model, small molecule metal-binding compounds can have metallochaperone activity by serving as a “sink and source” of zinc to facilitate coordination of zinc in its proper position and thus facilitate proper p53 folding. The key is choosing a metal-binding compound whose zinc affinity is less than that of the native p53 binding site. This allows the chelator to donate zinc to the native site. At the same time, the affinity must be stronger than that of the non-native binding sites to prevent zinc-induced misfolding. Up to now, this concept has only been demonstrated using purified WT DBD *in vitro*, and has never been demonstrated for full length, tetrameric p53 inside cells much less applied to restore WT structure of a missense mutant.

The relationship between p53 structure and zinc also applies to certain p53 mutants in which the mutation impairs zinc binding. This is best exemplified by p53-R175H. Studies reveal that this mutant is misfolded at physiologic temperatures due to an impairment in zinc-binding, resulting in the loss of site-specific DNA binding [11, 16]. It is hypothesized that this defect is present in mutants of the zinc-coordinating residues (C176, C238, C242, H179), but it is not known how many other mutants to which this may apply.

We hypothesized that ZMC1 restores WT structure and function to the p53-R175H by functioning as a zinc-metallochaperone to restore proper zinc binding. We aimed to determine if the model of zinc dependent folding and misfolding put forth by Loh and colleagues applies to p53-R175H and more broadly to other p53 mutants with impaired zinc binding. We also sought to determine if other zinc-binding small molecules could function as zinc-metallochaperones. Lastly we explored the role that redox changes induced by ZMC1 play in its mechanism of action.

## RESULTS

### Binding interactions of ZMC1, DBD, and Zinc

**Figure 1:**
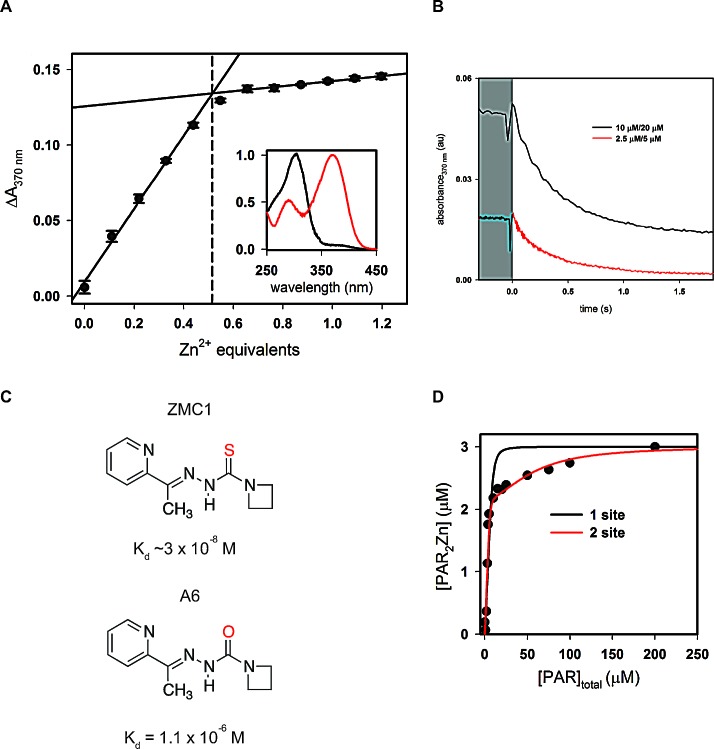
Binding interactions of ZMC1, DBD, and zinc A, titration of ZMC1 and ZnCl_2_. Extrapolation of the linear phases shows 0.51 ± 0.01 equivalents of Zn^2+^ at saturation. Inset shows normalized absorbance spectra for ZMC1 alone (black) and ZMC1-Zinc complex (red). B, measurement of ZMC1-zinc complex dissociation rate. Solutions of ZnCl_2_ and ZMC1 were mixed 1:1 with 2 mM EDTA to the final concentrations indicated (written ZnCl_2_/ZMC1). The traces are an average of 3-4 injections and when fit to a single exponential, yield nearly identical rates, which we combine to report a k_off_ of 2.6 ± 0.1 s^−1^. C, structures of ZMC1 and A6 and their K_d_'s for Zn^2+^. D, determination of the K_d_ of R175H DBD for Zn^2+^.R175H DBD (5 μM, 0.6 equivalents Zn^2+^ co-purified) was incubated with increasing concentrations of PAR to compete for the available Zn^2+^. The concentration of PAR_2_Zn^2+^ complex was determined by absorbance, and the parallel mass action and mass conservation equations solved to determine K_d_. K_d_'s from the 2-site model are 2.1 ± 0.8 nM for the tighter site (K_d1_) and below the detectable limit for the weaker site (K_d2_), respectively.

To define the binding interactions involved in ZMC1-mediated p53 reactivation, we sought to obtain K_d_ values of ZMC1 and zinc, DBD and zinc, and DBD and ZMC1. To measure the binding affinity of ZMC1 and zinc, we titrated a solution of ZnCl_2_ into 10 μM ZMC1 and monitored the change in absorbance at 370 nm (Fig. 1A). Binding is stoichiometric and therefore the K_d_ is too low to be measured at this concentration of ZMC1. However, extrapolating the linear portions of the curve to their point of intersection reveals that ZMC1 and Zn^2+^ form a 2:1 complex at saturation. To estimate the K_d_ we turned to kinetic methods. We measured the dissociation rate of the (ZMC1)_2_·Zn^2+^ complex by mixing it with a large excess of EDTA in a stopped-flow apparatus and monitoring the disappearance of the 370 nm absorbance peak (Fig. 1B). The off-rate (2.6 ± 0.1 s^−1^) fits to a single exponential function and is identical within error at two different ZMC1:ZnCl_2_ concentrations (5:2.5 μM and 20:10 μM), indicating that dissociation is a first-order process. Assuming a diffusion-limited association rate constant of 10^8^ (M ZMC1 dimer)^−1^s^−1^ yields a K_d_ of ~3 × 10^−8^ M for the reaction (ZMC1)_2_ + Zn^2+^⇆ (ZMC1)_2_·Zn^2+^.

For use in negative control experiments we synthesized compound A6 (Fig. 1C). A6 is identical to ZMC1 except it replaces the thiocarbonyl group with a carbonyl group to diminish its affinity for zinc. Experiments similar to those in Fig. 1A confirm that A6 binds Zn^2+^ ~100-fold weaker than ZMC1 (K_d_=1.1 × 10^−6^ M, Supplementary Fig. S1A). We further confirmed that A6 failed to inhibit cell proliferation or induce a WT-like conformation change in TOV112D (p53-R175H) detected by immunofluorescence (IF) as has been previously shown for ZMC1 (Supplementary Fig. S1B and C) [8].

To test the possibility that ZMC1 binds to DBD (WT or R175H) directly, we performed equilibrium dialysis between solutions of ZMC1:Zn^2+^ complex and protein. ZMC1 appears to accumulate slightly on the protein side for WT DBD but not enough to calculate aK_d_ (Supplementary Table S1). This putative interaction is weaker still in the case of R175H DBD. We estimate that if ZMC1 and DBD do interact, they do so with K_d_>10^−4^ M. Since ZMC1 induces apoptosis in cells at concentrations as low as 10^−8^ M, it is unlikely that the mechanism involves direct binding to DBD [8].

To determine the K_d_ between R175H DBD and Zn^2+^, we performed competition assays with the colorimetric zinc chelator 4-(2-pyridylazo)resorcinol (PAR). The affinity of WT DBD for zinc is too high to measure by this technique [11]. We incubated 5 μM R175H DBD, which purified with 0.6 molar equivalents of bound zinc, with increasing amounts of PAR and measured the concentration of PAR_2_·Zn^2+^at equilibrium by absorbance (Fig. 1C). The data cannot be modeled by zinc binding to a single site on R175H DBD. A minimum of two sites is required: a strong site (K_d1_) that accounts for one-third of the bound zinc, and a weak site (K_d2_) that accounts for the remaining two-thirds. The weak site binds zinc too weakly to compete with PAR, so we can only estimate a lower limit of K_d2_ ≥ 10^−6^ M. The strong site binds zinc tightly enough to compete with PAR, and fitting this portion of the curve yields K_d1_ = (2.1 ± 0.8) × 10^−9^ M. These data suggest that there are two populations of zinc bound to freshly purified R175H DBD. The majority of Zn^2+^ is bound to a weak, presumably incorrect site(s), and a minor fraction is bound to a high-affinity native site. The intracellular concentration of free Zn^2+^, estimated to be 10^−12^-10^−10^ M [17], is 10 – 1000-fold lower than K_d1_. We therefore conclude that R175H most likely exists in the cell in the zinc-free (apo) state, which explains its loss of WT function in cancer. That we can purify R175H with 0.6 equivalents of bound zinc, likely results from a combination of factors including rapid purification at low temperature, presence of trace amounts of Zn^2+^ in the buffers, and lack of chelators present during purification.

### ZMC1 rescues DBD from zinc-induced misfolding

**Figure 2:**
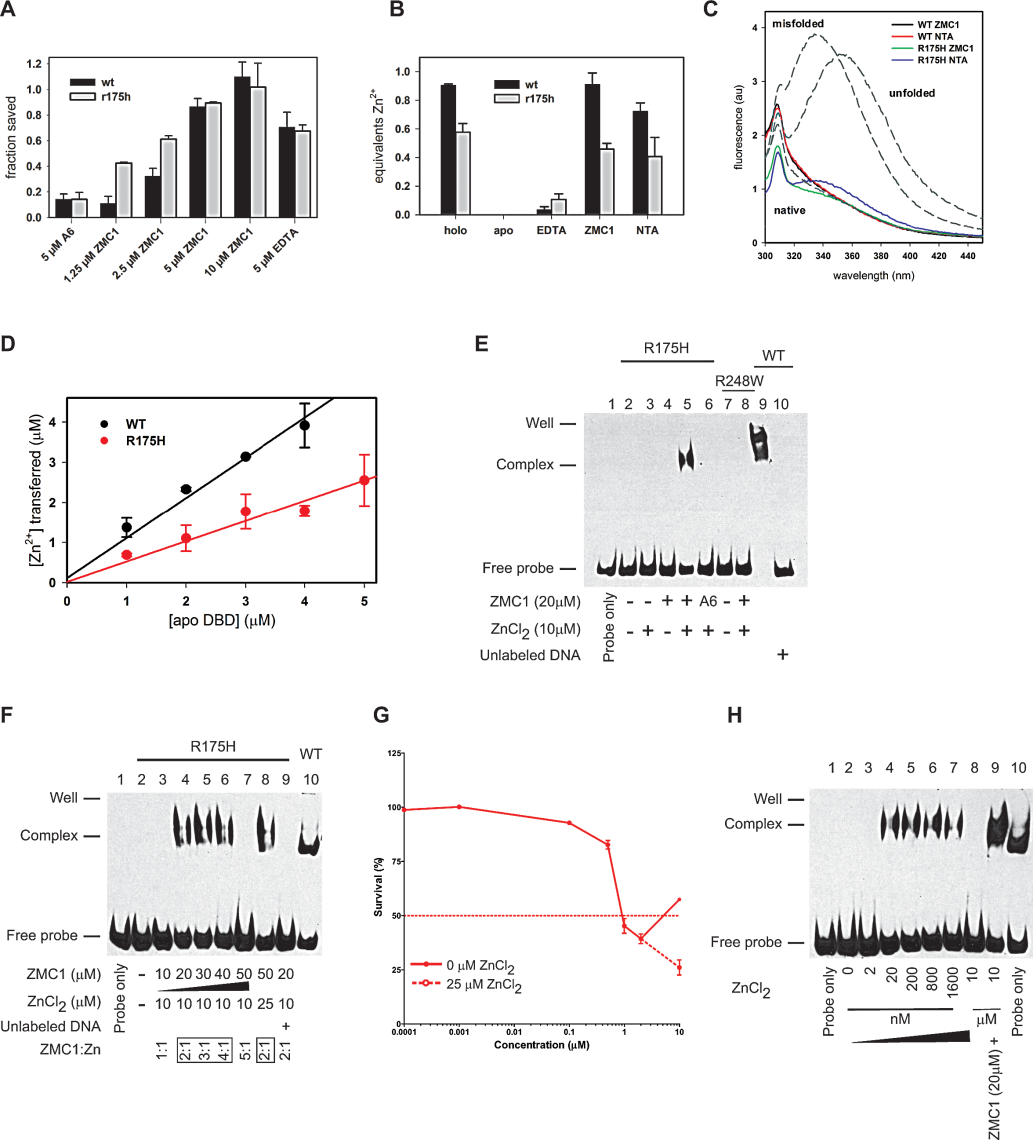
ZMC1 functions as a Zn-metallochaperone A, Trp fluorescence used to measure the fraction of R175H and WT DBD saved from Zn^2+^-arrested refolding. Values are normalized to no treatment and no ZnCl_2_ controls. Protein was unfolded in 5 M urea, refolded by rapid dilution in the presence of 2.5 μM ZnCl_2_, then rescued by the indicated treatment. Representative traces available in Supplementary Fig. S2. Error bars are ± SD. B, remetallationof WT and R175H DBD by Zn^2+^binding compounds. Apo DBD was incubated with ZnCl_2_ and either EDTA, ZMC1, NTA. The Zn^2+^ content of the resultant proteins was measured by PAR assay. ZMC1 and NTA can restore Zn^2+^ to pre-apoization levels. Error bars are ± SD. C, R175H and WT DBD remetallated by ZMC1 and NTA are native by Trp fluorescence. All spectra are of 0.5 μM protein desalted into buffer after the indicated treatment to remove any excess drug or zinc. Gray dashed lines are basis spectra from 0.5 μM WT DBD either freshly purified (native), unfolded in 8 M urea (unfolded), or misfolded with 5 μM ZnCl_2_ (misfolded). All spectra are similar to native DBD, with the exception of R175H remetallated with NTA, which is suggestive of a small amount of misfolding. D, quantification of Zn^2+^ transferred from ZMC1 to apo DBDs. The stoichiometries are 1.0 ± 0.1 equivalents (WT) and 0.5 ± 0.1 (R175H). Error bars are ± SD. E, electrophoretic mobility shift assay (EMSA) using WT, R175H and R248W DBD. The WT DBD is used as a positive control (Lane 9). Only the combination of ZMC1 (20μM) and ZnCl_2_ (10μM) restores site-specific DNA binding to the R175H DBD (Lane 5). The protein-DNA complex is specific because the unlabeled DNA competes for binding (Lane 10). F, EMSA demonstrating the importance of the stoichiometetry of ZMC1:Zn^2+^for the restoration of DNA binding. G, cell viability assay using serial dilutions of ZMC1. At 10 μM ZMC1, 25 μM ZnCl_2_ was supplemented. The ZMC1:Zn^2+^stoichiometric relationship applies to the pharmacodynamics of ZMC1 in p53-R175H cells. H, EMSA demonstrating that remetallation of the apo R175H DBD can occur in the absence of ZMC1. Free zinc of 2 nM to 10 μM and 20 μM ZMC1 were added to the indicated reactions.

We hypothesized that ZMC1 restores native structure and function to R175H DBD by functioning as a metallochaperone. This hypothesis stipulates that ZMC1 must prevent zinc from binding to non-native sites on DBD while simultaneously donating zinc to the native pocket. We performed the arrested folding assay to test the former. We previously showed that addition of more than one equivalent of free Zn^2+^ to urea-denatured WT DBD traps the protein in a misfolded state, preventing it from folding upon dilution of urea [15]. Misfolding is likely caused by zinc coordinating to one or more non-native sites that become available when the protein unfolds. We used Trp fluorescence to report on the conformational state of DBD. Unfolded DBD is identified by a peak centered at 355 nm, native DBD by an almost entirely quenched fluorescence spectrum, and misfolded DBD by a peak centered at 338 nm [15]. Addition of ZMC1 to WT and R175H DBD, previously arrested in their misfolded states by 2.5 μM ZnCl_2_, rapidly reverses misfolding and allows both proteins to resume folding at their normal rates (Supplementary Fig. S2). This effect is dose dependent with 100% rescue achieved at 10 μM ZMC1 (Fig. 2A). A6 has almost no effect on either protein. EDTA, which binds zinc much more tightly than ZMC1 (K_d_ = 8.1 × 10^−14^ M) yet is structurally dissimilar, is also able to rescue misfolding. This result indicates that ZMC1 and EDTA prevent misfolding via their common characteristic of zinc binding, consistent with our proposed metallochaperone mechanism [15]. However, our model predicts that ZMC1 will be able to restore function to R175H DBD, but not EDTA, because EDTA binds Zn^2+^>10,000-fold tighter than the K_d1_ of R175H DBD.

### ZMC1 restores native zinc binding to apoDBD

To test whether ZMC1 donates zinc to the WT and R175H DBD native binding sites and restores native structure, we incubated R175H and WT apoDBD (30 μM) with 60 μM ZnCl_2_ and 180 μM ZMC1 or 120 μM EDTA, and measured their zinc contents by PAR assay. EDTA does not remetallate either protein because it binds zinc too tightly (Fig. 2B). By contrast, ZMC1 is able to donate 0.91 (± 0.08) and 0.45 (± 0.04) equivalents Zn^2+^ per mole of WT and R175H DBD, respectively, which are identical within error to the zinc contents of the freshly purified proteins. The fluorescence spectra of the resultant proteins also resemble native protein (Fig. 2C). The above stoichiometry and the native-like fluorescence spectra of the remetallated proteins together suggest that ZMC1 delivers zinc to the native pocket while simultaneously preventing metal-induced misfolding.

To complement the remetallation assays of Fig. 2B, we used the optical properties of ZMC1 to observe the transfer of Zn^2+^from ZMC1 to the proteins in real time. We added 0–5 μM WT or R175H apoDBD to solutions of 15 μM ZMC1/5 μM ZnCl_2_ and monitored transfer of the metal from the drug to the protein by disappearance of the (ZMC1)_2_·Zn^2+^ absorbance peak at 370 nm. The transfer goes to completion in ~10 m regardless of the protein concentration used (Supplementary Fig. S3).We then used the amplitude of the absorbance change to quantify the amount of zinc transferred as a function of apo protein added (Fig. 2D). The data fit to lines with slopes of 1.0 ± 0.1 μM Zn^2+^/μM WT DBD and 0.5 ± 0.1 μM Zn^2+^/μM R175H DBD. These stoichiometries are in good agreement with those calculated by PAR assays (Fig. 2B), and that they are linear suggests that ZMC1 transfers zinc to a single site on each protein.

We used electrophoretic mobility shift assays (EMSA) to evaluate whether ZMC1 and zinc reactivate R175H apoDBD for site-specific binding to a DNA oligonucleotide bearing the p21 recognition sequence (Fig. 2E). The combination of 10 μM ZnCl_2_ and 20 μM ZMC1 (Lane 5) restored DNA binding to R175H apoDBD, but either one alone did not (Lane 3-4).20 μM A6/10 μM ZnCl_2_failed to induce a shift in the DNA (Lane 6) indicating the importance of zinc binding to ZMC1's activity. Furthermore, 20 μM ZMC1/10 μM ZnCl_2_failed to restore site-specific DNA binding to the DNA contact mutant R248W (Lane 7-8). The ZMC1-inducedDNA shift was sequence-specific as a mutant oligo containing 4/44 altered base pairs failed to undergo a shift (Supplementary Fig. S4).

To evaluate the relevance of the 2:1 ZMC1:Zn^2+^ stoichiometry to the restoration of DNA binding, we incubated R175H apoDBD with increasing concentrations of ZMC1 while holding the concentration of zinc constant at 10 μM (Fig. 2F). At 10 μM ZMC1 (1:1 molar ratio) we did not observe a DNA shift. Under these conditions the free zinc concentration is ~5 μM, which is greater than K_d2_ and therefore high enough to cause misfolding (Supplementary Fig. S2). By contrast, the combination of 10 μM ZnCl_2_ and 20 – 40 μM ZMC1 resulted in a shifted band. The shift is again lost at 50 μM ZMC1/10 μM ZnCl_2,_ probably because the free Zn^2+^concentration is reduced to the point where R175H DBD becomes starved of metal. In agreement, the shifted band reappeared when we increased the concentration of ZnCl_2_ to 25 μM (restoring the 2:1 ZMC1:Zn^2+^ ratio). These results suggest that ZMC1-mediated activation of R175H DBD is primarily dependent on the concentration of free zinc (rather than total zinc), which is determined in large part by the ratio of ZMC1 to Zn^2+^. Activation is likely to be robust over a wide range of total zinc concentrations as long as the ZMC1 concentration is maintained in moderate excess.

We next sought to determine if this relationship also applied in p53-R175H cells. Growth inhibition of TOV112D cells was maximal at 1-2 μM ZMC1, but decreased at 10 μM ZMC1 (Fig. 2G). We surmised that the decline in apoptosis was due to exceeding the optimal ZMC1:Zn^2+^ ratio. In support of this hypothesis, adding 25 μM ZnCl_2_ to the culture media restored growth inhibition and exceeded the inhibition seen in the 1-2 μM ZMC1 doses. We conclude the ZMC1:Zn^2+^ stoichiometric relationship is relevant to ZMC1 pharmacodynamics in cells.

Given the two-site binding model for zinc in R175H DBD (Fig. 1D), we predicted that R175H DBD could be converted to its functional zinc-bound form in the absence of ZMC1 if the concentration of free zinc was increased to greater than the K_d_ of the native site (K_d1_) but less than the K_d_ of the non-native site (K_d2_). We tested this using EMSA in which we could fine-tune the concentration of free zinc (Fig. 2H). We incubated R175H apoDBD with concentrations of ZnCl_2_ from K_d1_ (2 nM) to K_d2_ (>1 μM). We observed a shift at 20 nM ZnCl_2_, consistent with saturation of the native zinc binding pocket. The shifted band remained approximately constant up to 1.6 μM ZnCl_2_ then disappeared at 10 μM ZnCl_2_, likely because Zn^2+^ bound to the non-native sites and induced misfolding. Site-specific binding can then be restored to the sample containing 10 μM ZnCl_2_ if ZMC1 is added (Fig. 2G, Lane 9). These results validate our estimations for both K_d1_ and K_d2_ of R175H DBD made in Figure 1D. Furthermore, they indicate that remetallation of R175H does not require a zinc metallochaperone such as ZMC1, if the concentration of free zinc can be tightly controlled.

### Remetallation activity is not unique to ZMC1

**Figure 3:**
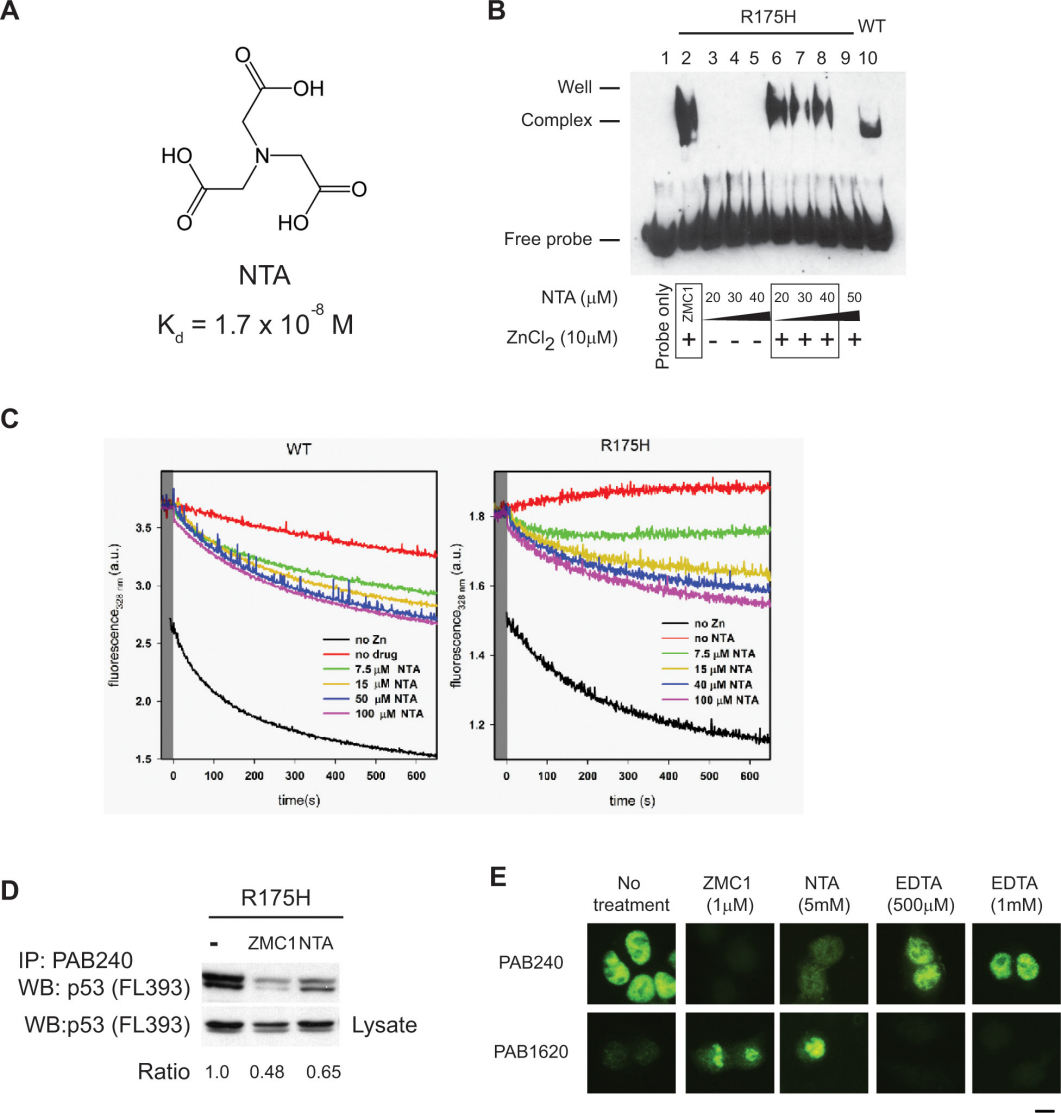
The zinc metallochaperone function is not unique to ZMC1 A, structure of NTA and its K_d_ for Zn^2+^. B, EMSA indicating R175H DBD binds to DNA in the presence of NTA and zinc with the same trend as ZMC1, but not with NTA alone. R175H DBD with ZMC1, zinc and WT DBD are used as positive controls. C, NTA arrested refolding traces. Experiments were run as in Figure S3 but with the indicated concentrations of NTA. NTA was effective at rescuing zinc induced misfolding, but less so than EDTA or ZMC1. D, immunoprecipitation (IP) of p53 protein from R175H cells after treatment of ZMC1 (1 μM) or NTA (5 mM) with the mutant specific PAB240 antibody. The density of western blot bands from IP and lysates were calculated and normalized to no treatment control. E, immunocytochemistry fluorescent staining (IF) of p53 protein from R175H cells after treatment of ZMC1 (1 μM), NTA (5 mM) or EDTA (500 μM or 1 mM). The antibody PAB1620 recognizes WT conformation of p53. Scale bar = 25 μm.

If ZMC1 can function as a zinc metallochaperone without interacting with p53, we reasoned its function would be shared by other compounds with similar zinc binding affinity. To test this hypothesis, we evaluated NTA, which binds zinc with a similar affinity to ZMC1 (K_d_ = 1.7 × 10^−8^ M), but is structurally unrelated (Fig. 3A). We incubated R175H and WT apoDBDs (30 μM) with 60 μM ZnCl_2_ and 180 μM NTA and measured their zinc contents by PAR assay. NTA was nearly as effective as ZMC1 in remetallating these proteins (Fig. 2B). However, a small amount of misfolding was noted in the samples remetallated with NTA suggesting that NTA does not protect against misfoldingas well as ZMC1 (Fig. 2C). We also tested NTA's ability to rescue DBD from zinc-arrested misfolding. NTA was able to rescue both WT and R175H DBD, but it required higher concentrations and was less effective compared to ZMC1 (Fig. 3C).

We next used EMSA to evaluate whether NTA can activate R175H apoDBD functionally. NTA alone (20-40 μM) did not restore DNA binding to R175H apoDBD, but did when combined with ZnCl_2_. NTA also exhibited the same stoichiometric relationship as ZMC1 in that a shift in DNA was seen for NTA:Zn^2+^ ratios of 2:1, 3:1 and 4:1, but was lost at 5:1 (Fig.3B). However, the bound oligonucleotide band is less pronounced than it is with ZMC1, indicating that ZMC1 is a more effective metallochaperone. As predicted by Fig. 2B, EDTA (20-300 μM) and ZnCl_2_ did not restore DNA binding activity by EMSA (Supplementary Fig. S5).

We then determined if NTA could restore WT conformation of R175H using cell-based assays. We incubated TOV112D cells with either ZMC1 or NTA and performed immunoprecipitation (IP) experiments with the antibody PAB240, which specifically recognizes misfolded and unfolded p53. Both ZMC1 and NTA substantially reduced the amount of misfolded R175H relative to the control (loss of 52% and 35% respectively) (Fig. 3D). Thus, both molecules induced a WT conformational change in p53-R175H, with ZMC1 being more effective than NTA in agreement with the EMSA experiments.

We substantiated these results by IF assays in TOV112D cells using both the native specific (PAB1620) and misfolded/unfolded specific (PAB240) antibodies. We previously demonstrated that the immunophenotype of the TOV112D cells is 1620-/240+ and that treatment with ZMC1 reverses this phenotype to 1620+/240-, indicating restoration of WT structure [8]. Both ZMC1 and NTA induced a reversal of the TOV112D immunophenotype to 1620+/240-, but EDTA did not (Fig. 3E). Taken together, our findings suggest that the zinc metallochaperone activity of ZMC1 is shared by other zinc-binding molecules with similar affinities for zinc.

### The ZMC1 zinc metallochaperone mechanism applies to other p53 missense mutants with impaired zinc binding

**Figure 4:**
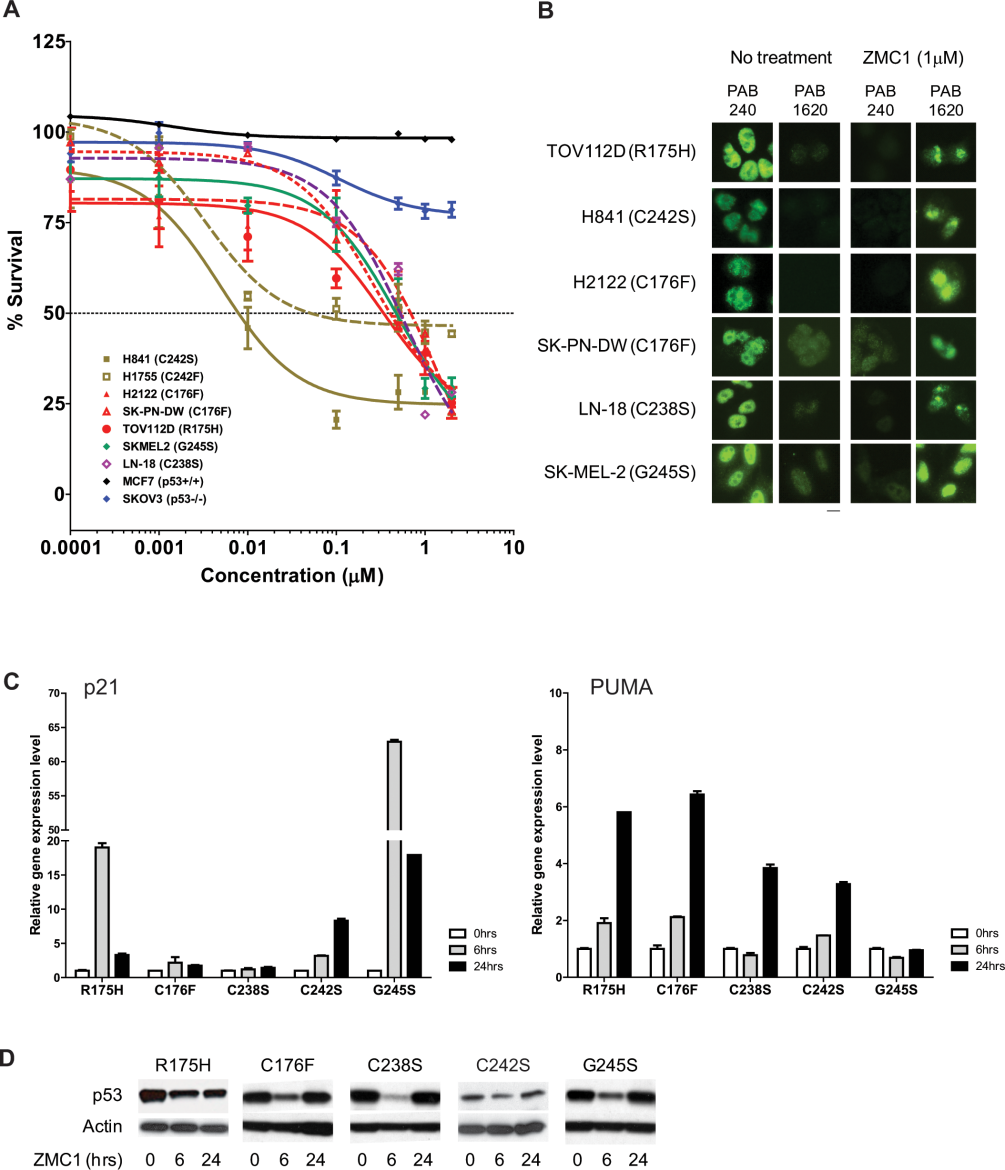
ZMC1 reactivates additional p53 missense mutants with impaired zinc binding A, ZMC1 inhibits cell growth in zinc-binding p53 mutants. Mutations are labeled in the legend. B, ZMC1 induces a WT like conformational change in zinc binding mutants, shown by IF. The antibodies, PAB240 and PAB1620 are same as Figure 3E. Scale bar = 25 μm. C, ZMC1 induces expression of p53 regulated genes (*p21* and *PUMA*) in zinc binding mutants, shown by quantitative RT-PCR. Relative gene expression level is normalized to β-actin. D, ZMC1 reduces mutant p53 stability in zinc binding mutants shown by western blot. The p53 protein level is detected by p53 antibody. β-actin is an internal control.

To investigate if ZMC1 can restore function to other p53 mutants with impaired zinc affinity, we tested the ability of ZMC1 to induce apoptosis in cell lines expressing mutations of the three zinc-binding Cys residues: C238, C242, and C176. H2122 and SK-PN-DW cells (p53-C176F) and LN-18 cells (p53-C238S) shared a similar sensitivity to ZMC1 (IC_50_ of 0.4-0.7μM compared to 0.37 μM for p53-R175H cells) (Fig. 4A). ZMC1 had less effect on MCF7 (p53-WT) and SKOV3 (p53-null) cells. H841 (C242S) and H1755 (C242F) cells were markedly more sensitive to ZMC1 (IC_50_ of 7.9 nM and 51 nM, respectively). To determine whether growth inhibition involves mechanisms other than p53 reactivation, we repeated these growth inhibition studies in the presence of an siRNA knockdown of mutant p53 in H841cells (Supplementary Fig. S6). This knockdown markedly attenuated the sensitivity of the cells to ZMC1 (IC_50 =_ 22 nM from 0.5 nM), but did not completely abrogate the effect, indicating a component of cell growth inhibition that was p53 independent as has been previously shown [8].

Like R175H, the C238S, C242F, and C176F mutations destabilize p53 and induce an unfolded/misfolded conformation that is recognized by the antibody PAB240. We next performed IF using the PAB1620/240antibodies on the above cell lines treated with ZMC1. ZMC1 treatment reversed the immunophenotype of p53 protein from 1620-/240+ to 1620+/240-, indicating that ZMC1 induces a WT-like conformational change in the C238S, C242F and C176F mutants (Fig. 4B). We also found in IP, ZMC1 reduced binding of PAB240 by 70% in H841 cells (Supplementary Fig. S6).

We then evaluated the restoration of p53 transcriptional activity by ZMC1 in C238S, C242F and C176F mutants by quantitating gene expression of p53 targets *p21* and *PUMA*. Similar to its effect on p53-R175H cells, ZMC1 potently induced *p21* and *PUMA* in these cells (Fig. 4C).

We previously showed that ZMC1decreases p53-R175H protein levels due to restoration of MDM2-mediated degradation [8]. Measuring p53 protein levels in ZMC1-treated cells is therefore a functional assay for p53 reactivation. ZMC1 treatment of p53-C238S, C242F, C176F cells resulted in a decline in p53 protein levels relative to the untreated control (Fig. 4D), supporting the conclusion that ZMC1 reactivates mutants of the three Cys involved in coordinating zinc.

We hypothesized that, like R175H, other mutants within the L2 or L3 loops of p53 may exhibit reduced zinc affinity and therefore be candidates for ZMC1 rescue. The X-ray crystal structure of WT DBD was recently solved to 2.05 Å resolution in the absence of DNA[18]. The authors concluded that no other amino acid besides the WT residue (Gly) could be substituted at position G245 without distorting the zinc binding site. Thus, we hypothesized that the G245S mutant might also be reactivated by ZMC1.

We found that G245S sensitivity to ZMC1 mirrored that of R175H and other zinc-binding mutants in cell growth inhibition assays (Fig. 4A). ZMC1 reversed the immunophenotype from 1620-/240+ to 1620+/240- shown by IF (Fig. 4B). Similar to the R175H mutant, we detected increased gene expression levels of *p21*, as well as decreased levels of the mutant protein upon ZMC1 treatment (Fig. 4C and). We therefore conclude that not only is the G245S mutant reactivated by ZMC1, but it can also be classified as one with impaired zinc binding. Alternatively, we examined another conformational mutant (R249M) that is recognized by PAB240 and did not find that this mutant was reactivated by ZMC1 (Supplementary Fig. S7). Thus we would conclude that the R249M conformational mutant likely does not have impaired zinc binding and that ZMC1 does not reactivate all conformational p53 missense mutants.

### ZMC1 transactivates the newly conformed mutant: a distinct component to the ZMC1 mechanism required for apoptotic function

We previously demonstrated that cellular glutathione levels were significantly reduced by ZMC1 indicating that ZMC1 affects the redox state of the cell [8]. We also demonstrated that the apoptotic function of ZMC1 could be inhibited by the co-administration of a reducing agent such as N-acetyl-cysteine (NAC) [8]. It is known that in response to cellular stress, p53 function is in part, mediated through post-translational modifications (PTMs) [19].

**Figure 5:**
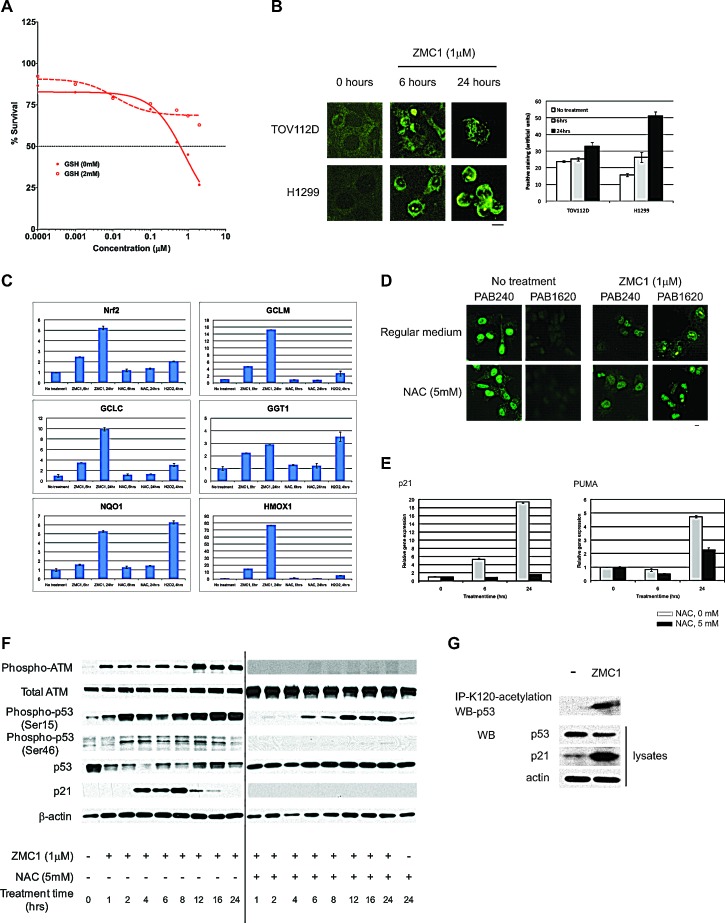
ZMC1 transactivates mutant p53 through increasing ROS levels A, exogenous GSH (2 mM) abrogates ZMC1 mediated cell growth inhibition. B, ZMC1 induces ROS levels in cells as detected by 8-oxy-dGUO staining. The right panel is quantification of staining. C, expression of Nrf2 and downstream targets is elevated after ZMC1 (1 μM) or H_2_O_2_ (300μM) but not NAC (5 mM) treatment. D, NAC (5 mM) does not inhibit ZMC1 mediated conformation change shown in IF. Scale bar = 25 μm. E, NAC (5 mM) abrogates ZMC1 mediated p53 transactivation in p53R175H cells and reduces expression of *p21* and *PUMA* genes. F, ZMC1 transcriptionally activates p53-R175H through ROS mediated post-translational modifications. NAC attenuates p53 post-translational modification induced by ZMC1. With addition of NAC, ATM, p53-Ser15 and Ser46 phosphorylation are reduced and p21 induction is eliminated. G, ZMC1 induces p53-K120 acetylation in p53-R175H cells.

We hypothesized that the ZMC1-induced redox stress functions to transactivate the newly restored mutant. To investigate this we first sought to confirm that the inhibitory activity of NAC on the apoptotic function of ZMC1 was mediated by an anti-oxidant function and not an alternate mechanism of NAC. Addition of another known anti-oxidant, glutathione (2 mM), significantly attenuated ZMC1 induced apoptosis (Fig. 5A). To confirm that ZMC1 induces ROS in cells, we performed IF on ZMC1 treated cells using an antibody that detects oxidized DNA (8-oxy-dGuo) on human tumor cell lines with different p53 status. We found that ZMC1 increased the 8-oxy-dGuo staining in both the p53-R175H and the H1299 (p53-null) cells at 6 and 24 hours (Fig. 5B). In addition, we found that ZMC1 treatment of p53-R175H cells significantly induced gene expression of the anti-oxidant transcription factor Nrf2 and several of its downstream targets (GCLM, GCLC, GGT1, NQO1, and HMOX1). Note that this induction could be abrogated by concomitant treatment with NAC (Fig. 5C).

We explored what role increased ROS levels play in the ZMC1 mechanism. It is possible that the ROS levels could be involved in the WT conformation change in p53-R175H cells as redox changes have been shown to induce conformation changes in WT p53 [20]. In the presence of NAC and ZMC1, the p53-R175H retained the WT conformation (1620+/240-) detected by IF (Fig. 5D). Thus NAC does not inhibit ZMC1 mediated apoptosis by interfering with the WT conformation change. However, NAC potently inhibited the ZMC1 mediated transcriptional upregulation of both *p21* and *PUMA* at 6 and 24 hours (Fig. 5E).

To determine if this inhibition was mediated by PTMs of the mutant p53, we examined the effects of ZMC1 alone and in combination with NAC on ATM activation and the phosphorylation status of serines 15 and 46 of p53 in p53-R175H mutant cells. Using a 24-hour time course western blot, we observed that ZMC1 treatment caused an activation of ATM that occurred within one hour after initiation of treatment and was sustained (Fig. 5F). This ATM activation is likely due to DNA damage as we also detected an increase in γ-H2AX levels by ZMC1 in H1299 cells that express either WT or R175H mutant p53 (Supplementary Fig. S8). ZMC1 also induced p53 phosphorylation at serines 15 and 46 (Fig. 5F). Note that mutant p53 levels decreased over time and then increased again with concomitant induction of p21 protein indicating reactivation of the mutant p53-R175H as had been previously shown (Fig. 5F) [8]. When NAC was included we found the following: 1) abrogation of ATM activation, 2) decrease in phosphorylation on serines 15 and 46 of mutant p53, 3) loss of p21 induction and 4) re-stabilization of mutant p53 levels (Fig. 5F). These results indicate that ZMC1 not only has a role in inducing a WT conformation of mutant p53, but that it also functions to transactivate the newly conformed mutant protein via PTMs indicative of WT p53. Acetylation of lysine at codon 120 has been shown to drive the p53 apoptotic response, which is the dominant cellular phenotype of ZMC1 treatment [21]. Indeed, treatment with ZMC1 induced K120 acetylation in p53-R175H mutant cells (Fig. 5G).

## DISCUSSION

**Figure 6:**
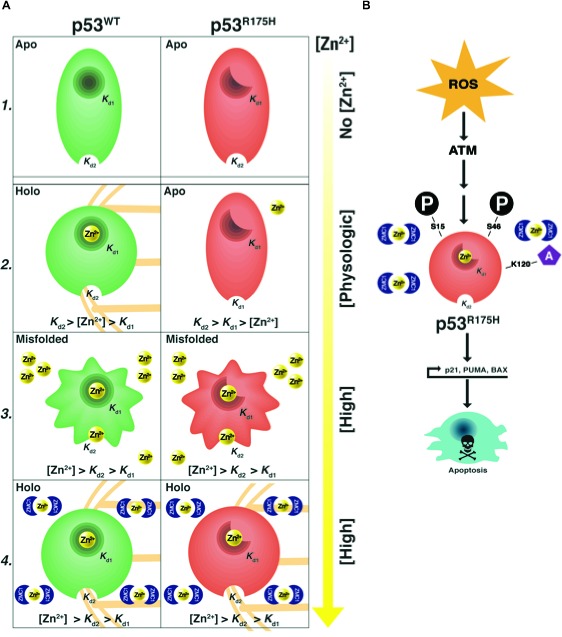
Dual mechanism of ZMC1 A1, in the absence of zinc both WT and R175H DBD are in the their zinc free (apo) forms. The K_d1_ of WT p53 is << K_d1_ of R175H p53 (≈2 nM). The K_d2_ of both WT and R175H p53 are ≥ 1 μM. A2, at physiologic concentrations of zinc, WT p53 is zinc bound (holo) (K_d1_<[Zn2+]<K_d2_). The R175H p53 is in its apo form ([Zn2+]<K_d1_<K_d2_). A3, in high concentrations of zinc, both WT p53 and R175H p53 are misfolded (K_d1_<K_d2_<[Zn2+]). A4, in the presence of ZMC1 both WT and R175H p53 are in their holo form despite (K_d1_<K_d2_<[Zn2+]). B, once the WT conformation change has occurred the increase in ROS by ZMC1 induces N-terminal post-translational modifications of mutant p53 (ser-15, 46, lys-120) that transactivate mutant p53 and induce an apoptotic program.

Our model for zinc-induced folding/misfolding and rescue by ZMC1 is shown in Fig. 6A. The model posits two types of zinc ligation sites on p53: native (K_d1_) and non-native (K_d2_) (Fig. 6A1). It is likely that multiple non-native sites exist, given that DBD contains 10 Cys and 9 His. PAR competition assays estimate K_d2_≥ 10^−6^ Μ for R175H (Fig. 1D) and we assume it to be the same for WT. K_d1_ of WT is too low to be measured by PAR but it was previously estimated to be less than 10^−10^ M [11]. For WT p53 to be functional in the cell K_d1_ must be in the pM range as the intracellular concentration of free zinc is typically maintained on the order of 10^−10^-10^−12^ M [17]. Thus, at physiological zinc concentrations, K_d1_ and K_d2_ of WT p53 are tuned for zinc binding to the correct site and not to the incorrect sites, and thus WT p53 is in its zinc-bound (holo) form (Fig. 6A2).

The situation is different for R175H. Its K_d1_ of 2 nM is 10 – 1000-fold higher than physiological zinc concentrations. R175H is therefore likely in the apo form in the cell (Fig. 6A2). Raising intracellular zinc above 2 nM can in principle reactivate R175H for DNA binding (Fig. 2G), but if levels rise beyond ~1 μM then R175H is expected to misfold due to non-native site zinc binding (K_d2_) (Figure 6A3). Thus at high concentrations of zinc (>1 μM) both WT and R175H mutant p53 are expected to be inactive as they would rapidly misfold (Fig. 6A3).

Metallochaperones such as ZMC1 and NTA are effective because they bind zinc less tightly than the native site and more tightly than the non-native sites. Indeed, their K_d_ values of 30 nM and 17 nM, respectively, lie in-between K_d1_ and K_d2_ of R175H DBD (Fig. 6A4). In this way, intracellular zinc concentrations can be buffered at levels optimal for restoring WT conformation to R175H. By contrast, EDTA and A6 possess K_d_ values less than K_d1_ and greater than K_d2_, respectively, and therefore are unable to function as metallochaperones.

In order for ZMC1 to rescue mutant p53 function *in vivo*, our model implicitly assumes that the drug increases the concentration of free Zn^2+^ in cells. It is possible that ZMC1 mobilizes intracellular stores of zinc from endogenous zinc-binding proteins such as metallothioneins. However, we regard it as more likely that additional zinc comes from sources outside the cell, including circulating serum which contains 8.5 – 23.6 μM Zn^2+^ as supplemental zinc increases the apoptotic function of ZMC1 [22]. It may prove beneficial to co-administer a zinc metallochaperone with supplemental zinc in patients.

The zinc-independent mechanism of ZMC1 pertains to the role of ROS. ZMC1 treatment elevates ROS levels, likely through iron chelation and Fenton chemistry as has been observed for other thiosemicarbazones [9]. These changes produce a stress response manifested by the activation of kinases such as ATM, resulting in PTMs of the mutant p53 N-terminus. These phosphorylation and acetylation events transactivate the protein and induce an apoptotic program making the drug therapeutic as a single agent (Fig. 6B).

The ROS signal that activates the newly conformed p53-R175H would presumably also be present in non-cancer cells and thus potentially activate WT p53. We did not find that ZMC1 increased p53 or p21 levels in several human tumor cell lines with WT p53 as was observed with the cytotoxic drug etoposide[8]. One reason this effect was observed in p53-R175H mutant cells may be that p53-R175H levels are much higher than that of WT p53 (which is relatively undetectable by western blot). Another may be that WT p53 cells are able to compensate for changes in ROS levels due to the anti-oxidant properties of p53 [23].

Our results indicate that ZMC1 activates mutant p53 by restoring proper zinc loading, rather than by conventional binding of drug to protein and altering its function/conformation. There are four lines of evidence for this conclusion. First, we did not detect a ZMC1-DBD interaction by equilibrium dialysis. Second, ZMC1 activity was reduced when the ZMC1:ZnCl_2_ ratio exceeded 5:1. If ZMC1 bound to p53 (either as the zinc-free or zinc-bound drug), increasing concentrations of ZMC1 would be predicted to increase its activity, not diminish it. Third, A6, which is structurally similar to ZMC1 but binds Zn^2+^ at least 100-fold weaker, fails to activate R175H p53. Lastly, NTA, acompound structurally unrelated to ZMC1 but with similar zinc affinity, also demonstrates zinc metallochaperone activity.

Evidence that mutant p53 could be reactivated by the addition of exogenous zinc was introduced by Puca et al. in which both the p53-R175H and p53-R273H mutants were reactivated by zinc supplementation [24]. Recently this group reported that a fluorescent zinc-curcumin complex could also reactivate the p53-R175H and R273H mutants; however the mechanism of this is not known [25]. It is unclear how p53-R273H can be reactivated by zinc as this is a DNA contact mutant which should not be able to bind DNA regardless of its zinc status. Nonetheless, our results provide the first evidence that WT structure/function can be restored to mutant p53 by a zinc metallochaperone. Pharmacologically, the strategy of delivering zinc by a small-molecule is preferable over administering exogenous zinc as our experiments indicate that the range of concentrations of free zinc required to remetallate the p53-R175H is quite narrow, and free zinc concentrations outside of this window quickly lead to misfolding. A zinc metallochaperone prevents this problem by serving as a zinc buffer.

Intriguingly, Puca et al. found that zinc supplementation was inadequate for p53 mediated tumor cell apoptosis, but rather it was necessary to co-administer a cytotoxic agent such as Adriamycin along with zinc to see mutant p53 induced cell death. These findings are consistent with our observations in that both restoration of WT structure and a transactivation signal are required. We observed both of these effects in ZMC1,which explains why the drug can be therapeutic as a single agent.

The broadening of the allele specificity of ZMC1 has important implications for drug development as it greatly increases the potential pool of patients that might benefit from synthetic zinc metallochaperones. The G245S mutant is the fifth most common p53 missense mutant accounting for 2.8% of p53 missense mutants [26]. When accounting for all of the known mutants with impaired zinc binding, we estimate this pool to be greater than 70,000 patients in the United States annually. Given that the G245S was not previously known to have impaired zinc binding, it also questions how many other conformational mutants share a similar defect. Thus the potential pool might be larger.

In conclusion, these findings illuminate a new path from which to drug mutant p53 using a small molecule acting as a zinc metallochaperone. From a broader perspective, the use of a compound to deliver a metal ion to correct a defect in protein folding is a novel mechanism for a small molecule therapeutic. Future studies will be necessary to illuminate the properties of a particular chelator that make it a good zinc metallochaperone. Our data indicate that one of these properties is its affinity for zinc. We speculate that both the zinc-metallochaperone and redox properties can be optimized through medicinal chemistry. This study also identifies the potential pool of patients that will most likely benefit from such a drug, which is a vital component to the successful development of any targeted molecular therapeutic. Our understanding of mutant p53 biology has grown considerably in recent years and we have come to appreciate that not all missense mutants are equivalent [26]. Our research here provides a practical need to discriminate these mutants in the application of mutant p53 based cancer therapeutics.

## METHODS

### Biochemical Analysis

Proteins were prepared as previously described with minor modifications detailed in Supplemental Methods [27]. ZMC1-Zn^2+^ titration, competition binding, equilibrium dialysis, arrested refolding, Zn^2+^ content measurement, re-metallation analysis, Zn^2+^ transfer measurements, stopped-flow kinetics, and EMSAs were conducted using standard protocols indicated in the text and are detailed in the Supplementary Methods.

### Synthesis of compound A6

Details of A6 synthesis are found in the Supplementary Methods. The compound was analyzed with LC/MS MH+ 219, 97% pure. Structure determined with Bruker 400 mHz NMR (1H NMR, 13C NMR, HSQC).

### Cell culture and cell-based analysis

TOV112D, SK-PN-DW, LN-18, SK-MEL-2, H1299 were cultured in DMEM with 10% FBS. MCF7, H841, H2122, H1755, Hs700T were cultured in RPMI with 10% FBS. SKOV3 and HCT116 were cultured in McCoy's 5A with 5% FBS. Treatment, viability assays, transfection, immunofluorescent staining, immunoprecipitation, RNA extraction and quantitative RT-PCR, and western blots were conducted using standard procedures detailed in the Supplementary Methods.

## SUPPLEMENTARY MATERIAL FIGURES AND TABLE




