# Supplementary material for: Small molecule restoration of wildtype structure and function of mutant p53 using a novel zinc-metallochaperone based mechanism
Source: Oncotarget. 2014 Sep 3;5(19):8879–92. doi: 10.18632/oncotarget.2432 (PMC4253404; doi:10.18632/oncotarget.2432)
Supplement: Supplementary file 1 [file oncotarget-05-8879-s001.pdf]

Small molecule restoration of wildtype structure and function of mutant p53 using a novel zinc-metallochaperone based mechanism

Supplementary Material

Protein preparation

There were two sources of p53 (94-312) DNA Binding Domain (DBD) used in this manuscript. His-tagged preps of all DBD mutants used were provided by Novartis. Preps without affinity tags were prepared in-house. Experiments were conducted with both sources and yielded similar results. The in-house proteins were expressed and purified as described previously with minor modifications [1] DBD was expressed in Escherichia coli strain BL21(DE3) (Novagen). Cells were grown to OD_{600} = 0.6 at 30 ˚C before induction with 15 mg/L Isopropyl β-D-1-thiogalactopyranoside and overnight growth in a refrigerated shaker at 18 ˚C. Cells were harvested by centrifugation and resuspended in 50 mM Tris pH 7.2 and 20 mM β-mercaptoethanol (β-ME). Cells were disrupted enzymatically by egg white lysozyme (Sigma-Aldrich) and DNAase1 from bovine pancreas (EMD Millipore) on ice, and DBD purified by sequential SP-Sepharose cation-exchange and heparin pseudo-affinity chromatography of the soluble lysate as described (Bullock et al 1997). Proteins were judged to be >98% pure by SDS-PAGE stained with Coomasie brilliant blue. Protein concentrations were determined by absorbance using ϵ_{280} = 17,190 $M^{-1}cm^{-1}$ [2]. Apo proteins were generated as described previously [1]. All experiments were performed in 50 mM Tris, 0.1 M NaCl, and 1 mM tris(2-carboxyethyl)phosphine hydrochloride (TCEP) unless otherwise noted.

Spectroscopic measurements

Absorbance experiments were performed in a Varian Cary-100 UV-Visible spectrophotometer equipped with thermostated cuvette holder. Fluorescence experiments were performed on a

Fluoromax-4 spectrofluorimeter equipped with thermostated cuvette holder (Jobin-Yvon). Fluorescence measurements were taken in a 1 cm by 1 cm quartz cuvette with excitation and emission slits of 3 nm and 4 nm respectively. All experiments were conducted at 10 ˚C unless otherwise noted.

ZMC1 - Zn^{2+} binding stoichiometry titration

$ZnCl_2$ was titrated into a solution of 10 μM ZMC1 at room temperature (RT) and the absorbance at 370 nm monitored. The absorbance values were corrected for dilution, and the rise and plateau phases of the curve were fitted with lines. The point of intersection was used to determine the saturating stoichiometry of Zn^{2+} to ZMC1.

Competition binding

Freshly purified R175H (5 μM), which co-purified with 0.6 equivalents of bound Zn^{2+}, was incubated with increasing concentrations of 4-(2-pyridylazo)resorcinol (PAR, Sigma-Aldrich) and left to equilibrate at 10 ˚C for 1 h. The concentration of PAR_2Zn complex was then measured by absorbance at 500 nm. Samples were blanked against equivalent PAR solutions without protein added. No additional Zn^{2+} was released from the protein when denatured in 6 M guanidine-hydrochloride (Gdn-HCl), so we concluded that all available Zn^{2+} was accounted for in our competition binding curve. The K_d was determined by solving the following mass action and mass conservation equations in parallel as described[3], followed by least squares minimization using a custom script written in MATLAB R2010a (MathWorks):

(1) $K_d = [P]_{free} * [Zn]_{free} / [PZn]$

(2) $\beta_2 = [PAR_2Zn] / ([PAR]^2_{free} * [Zn]_{free})$

(3) $[P]_{total} = [P]_{free} + [PZn]$

(4) $[Zn]_{total} = [Zn]_{free} + [PZn] + [PAR_2Zn]$

(5) $[PAR]_{total} = [PAR]_{free} + 2 * [PAR_2Zn]$

Where K_d is the dissociation constant of the protein for Zn^{2+}, $[P]_{free}$ is the concentration of apo protein, $[Zn]_{free}$ is the free Zn^{2+} concentration, [PZn] is the concentration of Zn^{2+} bound protein, β_2 is the equilibrium constant between the PAR_2Zn complex and the free species (10^{12} M^{-2}) [4], $[PAR_2Zn]$ is the measured concentration of the PAR_2Zn complex, $[PAR]_{free}$ is the concentration of free PAR, $[P]_{total}$ is the known total concentration of protein, $[Zn]_{total}$ is the known total concentration of Zn^{2+}, and $[PAR]_{total}$ is the known total concentration of PAR.

Equilibrium Dialysis

Equal volumes of DBD at ~20 μM and a mixture of 20 μM/10μM ZMC1 and $ZnCl_2$ were loaded onto either side of an acrylic microdialysis chamber separated by a piece of 8000 MWCO dialysis tubing (BioDesignInc). The membrane was pretreated by boiling in 2% sodium bicarbonate buffer and 1 mM EDTA for 10 min to remove any excess Zn^{2+} and rinsed thoroughly. Samples were shaken vigorously for 24 hrs, and the concentration of $ZMC1_2Zn$ complex on each side of the membrane was determined by absorbance at 370 nm using $\epsilon_{370} = 1.4 \times 10^4$ $M^{-1}cm^{-1}$ (where each concentration unit represents 1/2 of a $ZMC1_2Zn$ complex) as determined by our lab.

Arrested refolding

Arrested refolding experiments were performed as described previously with minor modifications [5]. DBD (25 μM) was denatured in buffer containing 5 M urea, and then rapidly diluted 50-fold into urea-free buffer with or without 2.5 μM $ZnCl_2$ to final concentrations 0.5 μM DBD and 0.1 M urea with continuous magnetic stirring. Intrinsic tryptophan fluorescence at 328 nm was monitored (excitation 280 nm). For $ZnCl_2$-containing samples, after ~90 s of stalled-folding, the indicated concentration of EDTA, NTA, ZMC1, or A6 was added and folding monitored as before. Kinetic traces were corrected for inner-filter effect empirically. The endpoints of the curves were normalized to samples with no drug added and no $ZnCl_2$, and used to calculate the fraction of protein rescued from misfolding.

Zn^{2+} content measurement

DBD samples were desalted using a PD-10 column (Amersham) equilibrated 50 mM Tris pH 7.2, 150 mM NaCl, and 1 mM TCEP. Samples (1-10 μM) were then unfolded in 6 M Gdn-HCl and reacted with 1 mM N-ethylmaleimide for 20 m at RT to ensure complete release of all bound Zn^{2+}. The reaction was quenched with 10 mM β-ME for 10 min, and Zn^{2+} concentration measured by PAR assay using $\epsilon_{500} = 5.0 \times 10^4$ for the PAR_2Zn complex in buffer containing 6 M Gdn-HCl as determined by our lab. Measured Zn^{2+}concentration was divided by protein concentration to determine Zn^{2+} content of the protein.

Re-metallation

After apoization, DBD (30 μM) was incubated with a mixture of 60 μM $ZnCl_2$ and 120 μM EDTA, 180 μM ZMC1, or 180 μM NTA for 20 min on ice. Samples were then desalted and Zn^{2+} content assayed as above.

Zn^{2+} transfer kinetics

ZMC1-Zn complex was pre-formed by mixing 15 μM ZMC1 with 5 μM $ZnCl_2$, followed by the addition of 0-5 μM apo DBD. Transfer of Zn^{2+} from ZMC1 to DBD was monitored by absorbance 370 nm. The amount of Zn^{2+} transferred was calculated by the amplitude of the signal change (each Zn^{2+} transferred is 2 units of ZMC1 concentration using $\epsilon_{370} = 1.4 \times 10^4$ $M^{-1}cm^{-1}$). The concentration of Zn^{2+} transferred was then plotted as a function of DBD concentration and fit with a line to determine the stoichiometry of the transfer.

Synthesis of compound A6

Azetidine (85 mg, 1.5 mmol) was dissolved in 5 mL methylene chloride and diisopropylethylamine (195 mg, 1.50 mmol, 1.0 equiv.) was added followed by triphosgene (267 mg, 0.9 mmol, 0.6 equiv.) and the reaction mixture stirred at ambient temperature for 4 hrs to generate the azetidine-1-carbonyl chloride in situ. Without workup, (E)-2-(1-hydrazonoethyl pyridine (203 mg, 1.5 mmol, 1.0 equiv.) was added directly to this mixture following by additional diisopropylethylamine 195 mg, 1.50 mmol, 1.0 equiv.) and the reaction continued to stir at ambient temperature for 72 hrs. The reaction was then evaporated to dryness and purified by silica gel chromatography (high purity grade silica gel, 32-64 microns, 230-400 mesh) eluted with a gradient of 5-10% methanol/methylene chloride with 1% triethylamine to give a white

crystalline solid in selected middle fractions. These crystals were washed with 5:1 hexanes:methylene chloride, and dried under vacuum to give 24 mg of (*E*)-*N'*-(1-(pyridin-2-yl)ethylidene)azetidine-1-carbohydrazide (A6) as a free flowing white crystalline solid. Analysis of A6 was analyzed with LC/MS MH+ 219and showed 97% pure. Structure determined with Bruker 400 mHz NMR (1H NMR, 13C NMR, HSQC).

A6 K_d measurement

$ZnCl_2$ was titrated into a solution of 5 μM A6 at RT and the absorbance change at 360 nm monitored. The resultant curve was then fit to the following equation in KaleidaGraph 4.03 (Synergy Software):

$$\Delta A = \Delta A_{max} * (([D] + [L] + K_d) \pm \mathrm{sqrt}(([D] + [L] + K_d)^2 - 4 [D] [L])) / (2 [D])$$

Where ΔA_{max} is the absorbance change at saturating $ZnCl_2$, [D] 1/2 the total concentration of A6 (as required by the 2 to 1 binding mechanism), [L] is the concentration of $ZnCl_2$, and K_d is the dissociation constant for drug and ligand.

Stopped-Flow Kinetics

Rapid kinetic measurements were carried out on a Bio-Logic SFM4-Q/S stopped-flow device. The mixing dead-time was ~2 ms. Absorbance was observed at 370 nm with a 2 nm bandpass. At room temperature, pre-mixed solutions of ZMC1 and $ZnCl_2$ were mixed with an equal volume of buffer to yield the indicated concentrations and observed for 200 ms to establish a baseline. They were then mixed with an equal volume of buffer containing 2 mM EDTA and the

dissociation observed. Traces shown are an average of 4 injections in series. Kinetic traces were fit to a single exponential in Bio-Kine32 (BioLogic).

Electrophoretic mobility shift assay (EMSA)

EMSA were performed as follows. Purified p53 DBDs (WT, R175H, R248W) were used in the indicated reactions. Various concentrations of ZMC1, A6, $ZnCl_2$, NTA or EDTA were used in the reactions. DNA was 44-bp p53 recognition sequence (p53RE) in the promoter sequence of p21 gene [6], 5' F-AGCTAGTAGAGCGAACATGTCCCAACATGTTGGCGTGCTGCAGC, 5' R-GCTGCAGCACGCCAACATGTTGGGACATGTTCGCTCTACTAGCT. The 4 nucleotide mutations were introduced to make mutant DNA sequence (labeled as lowercases), 5' mut F-AGCTAGTAGAGCGAAtATaTCCCAAtATaTTGGCGTGCTGCAGC and 5' mut R-GCTGCAGCACGCCAATATATTGGGATATATTCGCTCTACTAGCT. DNA was labeled with biotin, using DNA 3' End Biotinylation Kit (Thermo Fisher Scientific/Pierce). Unlabeled DNA was used to compete the binding. Reaction mixture was incubated at 4 ˚C for 10 min prior to addition of biotin labeled probes. Binding buffer was described before [6]. DNA binding reactions were carried out at 4 ˚C for 20 min, and resulting DNA-protein complexes were separated on a 6% polyacrylamide gel (made with 30% Acrylamide/Bis Solution (37.5:1) (Bio-Rad)) in 0.5× Tris-borate (without EDTA), running in 0.5× Tris-borate (without EDTA) buffer. Complexes were visualized by LightShift Chemiluminescent EMSA Kit (Thermo Fisher Scientific/Pierce). NTA and EDTA were from Sigma.

Cell lines and culture conditions

TOV112D, SK-PN-DW, LN-18, SK-MEL-2, H1299 were cultured in DMEM with 10% FBS. MCF7, H841, H2122, H1755, Hs700T were cultured in RPMI with 10% FBS. SKOV3 and HCT116 were cultured in McCoy's 5A with 5% FBS. GSH and NAC were from Sigma-Aldrich.

Viability assay

Viability assays were done according to the manufacture instruction of Guava ViaCount (Millipore). In brief, the cells ($5x10^4$ cells/well) were cultured in a 12-well plate to reach the 50-60% confluence on the second day when treated with serial dilutions of the compound. Cell growthwas measured by Guava ViaCount reagent and Guava PCA instrument after incubation for 3 days.

Transfection of p53 siRNA

The siRNA transfection is done with Lipofectamine 2000 (Invitrogen), following the manufacture's instuctions. The p53 siRNA was from SMARTpool (Thermo Fisher Scientific/Dharmacon).

Immunofluorescent staining

The cells were grown on the coverslip, followed by various treatments. The coverslips were fixed with 4% paraformaldehyde for 10 min and then permealized with 0.5% Triton-X100 for 5 min. The conformation of the mutant and WT p53 proteins were recognized specifically by the antibodies PAB1620 (1:50, recognizing WT conformation) and PAB240 (1:200, recognizing

mutant conformation) stained overnight, respectively. The secondary antibody, goat anti-mouse IgG was incubated for 40 min. PAB1620 and PAB240 were from EMD Chemicals.

Immunoprecipitation (IP)

The cell lysates (500 μg) with various treatments were subjected to immunoprecipitation with ImmunoCruz Optima Immunoprecipitation reagent (Santa Cruz Biotechnology) using the antibody PAB240 (4 μg). The pulldown was detected by western blot with p53 (FL393) (Santa Cruz Biotechnology) that recognizes all formats of the p53 protein. The density of the image relative to the Control was determined using Adobe Photoshop.

RNA extraction and quantitative RT-PCR

RNA was extracted from the cells using RNeasy kit (Qiagen) and the gene expression level was measured by quantitative RT-PCR using TaqMan gene expression assays (Life Technologies/Applied BioSystems). The gene expression level was normalized with β-actin and the average was presented with standard deviation from triplicates of repeated experiments.

Western blot

The lysates (or immunoprecipitated products) were run on SDS-PAGE and transferred onto PVDF membranes. The detection of the protein level was done with the manufacture instruction (Western Lightning® Plus-ECL, Perkin-Elmer). The p21 antibody was from EMD Chemicals. The actin antibody and the p53 (DO-1 and FL393) were from Santa Cruz Biotechnology. Phospho-ATM, total ATM, Phospho-p53 (Ser15) and Phospho-p53 (Ser46) antibodies were from Cell Signaling.γ-H2AX antibody was from Abcam. p53-K120 acetylation was from Millipore.

The density in IP was determined using Adobe Photoshop and was expressed as the ratio of IP band to lysate band then relative to the control (no treatment).

8-oxy-dGUO staining

8-oxy-dGUO antibody was from Trevigen. The immunocytochemistry staining was followed the manufacturer's instruction.

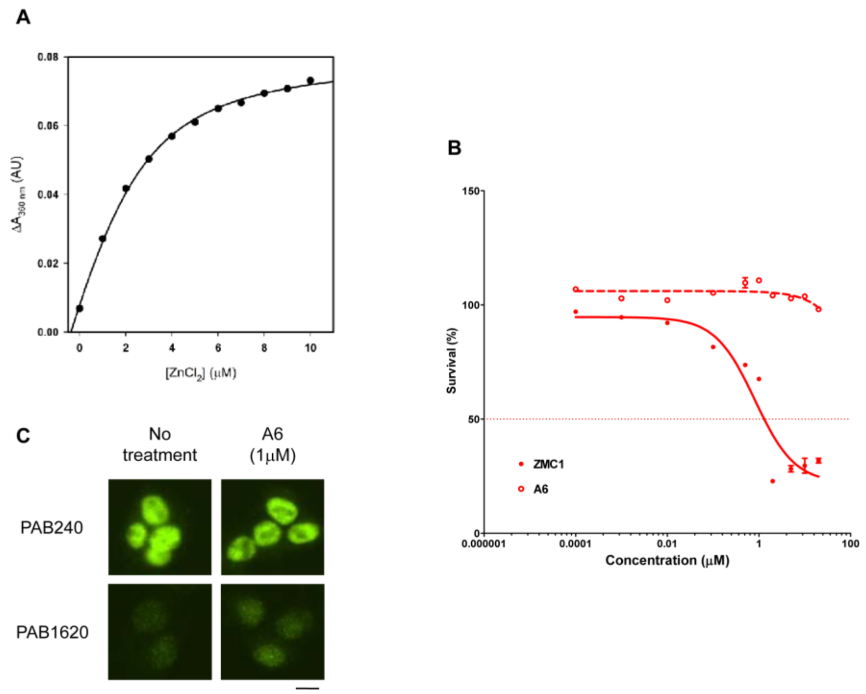

Figure S1: A6, ananalog of ZMC1, lacks zinc binding and does not function as a ZMC1. **A,** K_d measurement of A6 for Zn^{2+}. $ZnCl_2$ was titrated into a solution of 5 μM A6 in buffer, which yielded a change in absorbance similar to ZMC1 with a peak at 360 nm. The results were fit to the equilibrium binding equation assuming the same 2:1 binding mechanism as ZMC1, which yields a $K_d = 1.1 \pm 0.1$ μM, ~100-fold weaker than that estimated for ZMC1. **B,** cell viability assay using serial dilutions of ZMC1 or A6. The cells growed in 12-well plates and treated for 3 days followed by measurement of viabiltiy using Guava ViaCount. Solid line was ZMC1 treatment, and dashed line was A6 treatment. **C,** immunofluorescent staining of p53 protein after treatment of A6 (1 μM) using PAB240/PAB1620. Scale bar = 25 μm.

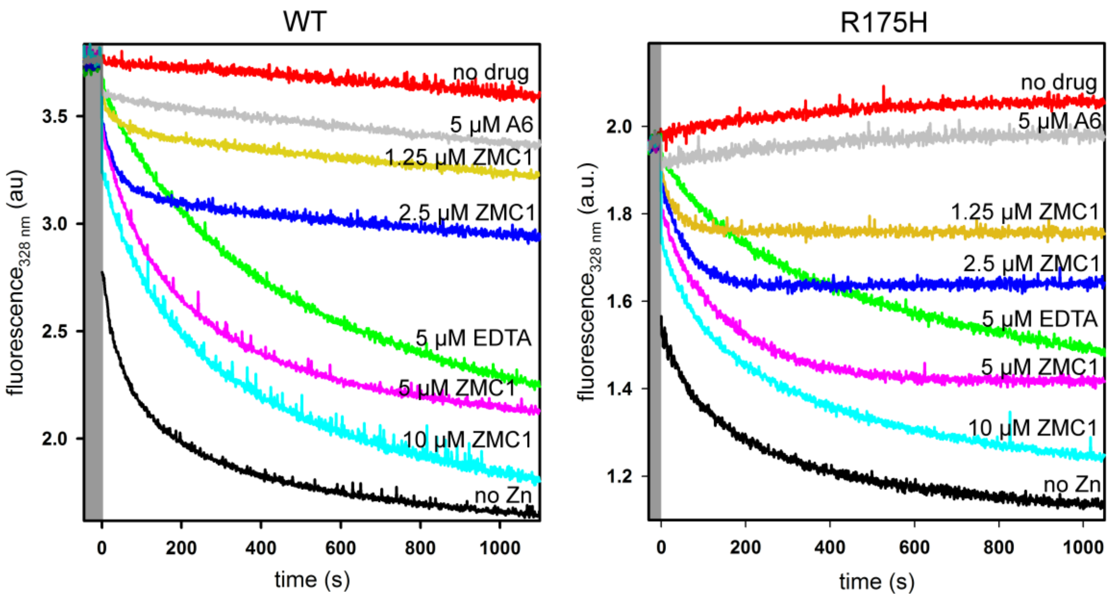

Figure S2: ZMC1 rescues WT and R175H DBD from zinc induced misfolding. Samples of WT and R175H DBD were unfolded in 5 M Urea, then diluted into buffer to the final concentrations of 0.5 µM protein, 0.1 M Urea, and 2.5 µM $ZnCl_2$ at 10˚C. After ~90 seconds, the indicated treatment was added (time 0). The disappearance of the misfolded fluorescence peak was monitored at 328 nm (the isosbestic point between ZMC1 alone and the ZMC1-Zn^{2+} complex) to minimize changes in inner filter effect as ZMC1 bound more Zn^{2+}throughout the experiment. The traces shown were corrected empirically for inner filter effect. The grey box represents the arrested phase of the experiment. The endpoints of similar traces were used to quantify the fraction saved graph shown in Figure 2A.

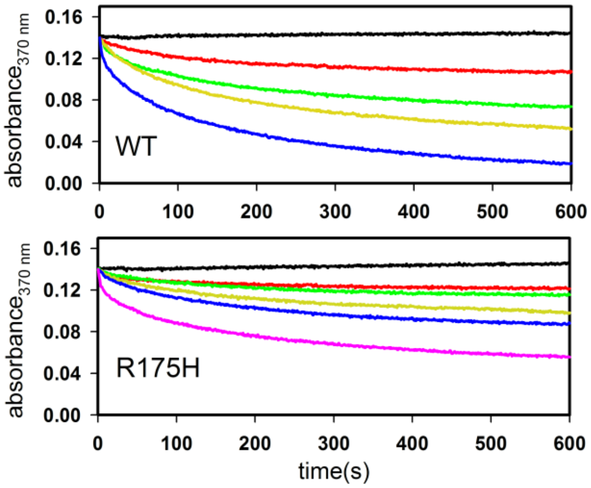

Figure S3: ZMC1 transfers zinc to apo R175H and WT DBD in real time. Increasing concentrations of apo DBD were added to buffer containing 5 µM $ZnCl_2$ and 15 µMZMC1 at 10˚C, and the absorbance at 370 nm was monitored over time. The traces shown are 0 µM (black), 1 µM (red), 2 µM (green), 3 µM (gold), 4 µM (blue), 5 µM (magenta). ZMC1 was able to release Zn^{2+} to the protein at a consistent stoichiometry in a matter of minutes.

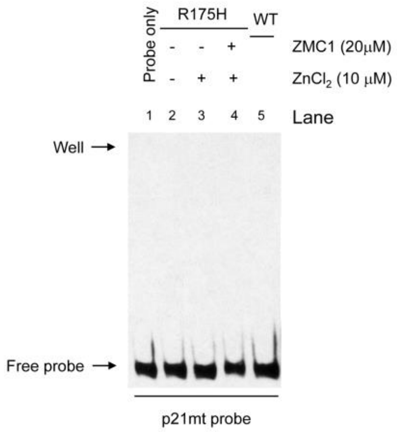

Figure S4: The binding of p53 DBD to the p53 response element (p53RE) in the p21 gene is sequence specific. Electrophoretic mobility shift assay (EMSA) using WT and R175H and mutant p53RE in p21 promoter. The sequence was described in the Supplemental Methods.

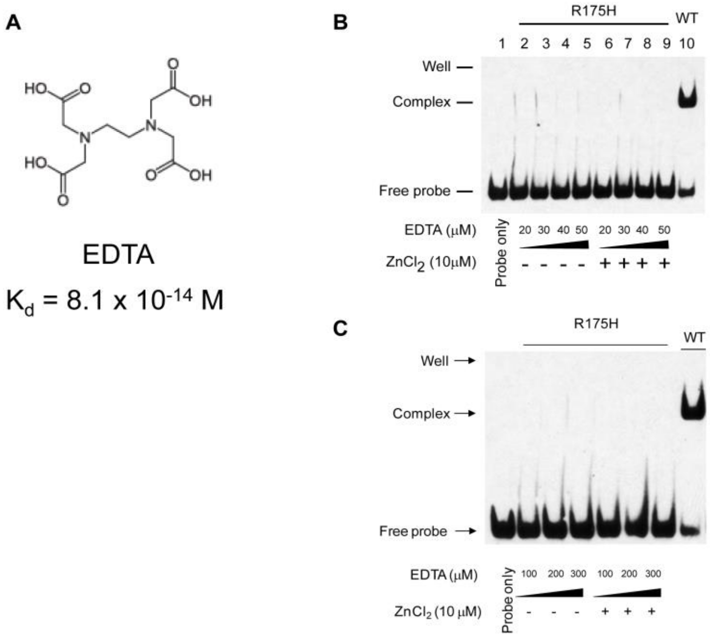

Figure S5: R175H DBD does notbind DNA with EDTA with or without zinc, even at higher concentrations of EDTA. **A,** structures of EDTA and its K_d for Zn^{2+}. **B,** EMSA was done with R175H DBD in the presence of EDTA (20-50 μM) and/or zinc. WT DBD was used as a positive control. **C,** EMSA was done with R175H DBD in the presence of EDTA (100-300 μM) and/or zinc. WT DBD was used as a positive control.

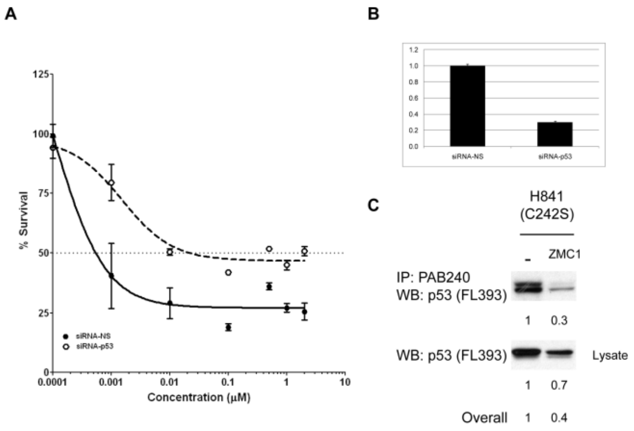

Figure S6: The p53 C242S mutantis more sensitiveto ZMC1 than the R175H mutantand it is p53 dependent. **A,** Cell viability assay using cells with and without transfection with siRNA to p53. Solid line was cells with transfection with siRNA to non-specific sequence (siRNA-NS), and dashed line was cells with transfection with siRNA to p53 (siRNA-p53). **B,** Quantitative RT-PCR of the p53 gene expression level in the cells with transfection to show p53 knockdown efficiency by siRNA. **C,** Immunoprecipitation (IP) of p53 protein from C242S cells after treatment of ZMC1 (1 μM). The IP antibody, PAB240, specifically recognizes mutant p53 protein. The density of western blot bands from IP and lysates were calculated and normalized to no treatment control.

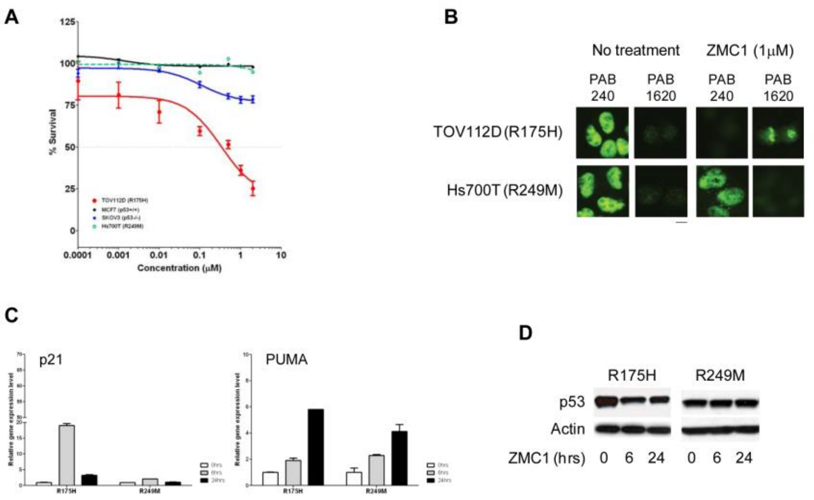

Figure S7: ZMC1 does not reactivate conformational mutant p53-R249M. **A,** ZMC1 did not inhibit cell growth in zinc-binding p53 mutants. Mutations are labeled in the legend. **B,** ZMC1 did not induce a WT like conformational change in zinc binding mutants, shown by IF. The antibodies, PAB240 and PAB1620 are same as Figure 3E. Scale bar = 25 μm. **C,** ZMC1 did not induce expression of p53 regulated genes (*p21* and *PUMA*) in p53-R249M, shown by quantitative RT-PCR. Relative gene expression level is normalized to β-actin. **D,** ZMC1 did not reduce mutant p53 stability in p53-R249M shown by western blot. The p53 protein level is detected by p53 antibody. β-actin is an internal control.

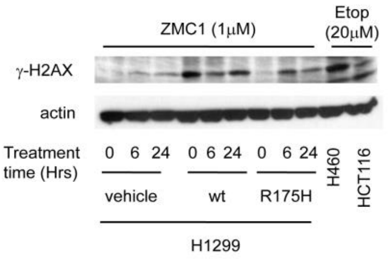

Figure S8: ZMC1 induces γ-H2AX in the cells.H1299 (p53-null) cells were transfected with expression vectors for p53-R175H or WT p53 and treated with ZMC1 (1 μM, 6 and 24 hrs). γ-H2AX levels detected by western blot indicate detection of DNA damage by increases in ROS. The cells lines bearing WT p53 (H460 and HCT116) treated with etoposide (Etop) (20 μM) were used as positive controls.

Table S1. Equilibrium dialysis reveals minimal binding between DBD and ZMC1. 20 μM ZMC1 + 10 μM $ZnCl_2$ was dialyzed against the indicated concentration of protein. Every unit of concentration represents 1/2 of a 726-Zn complex measured by absorbance at 370 nm. Higher concentrations were attempted but failed as the drug would stick to the equilibrium dialysis vessels.

A

r175h (19.3 μM)

Trial #	[Drug-Zn] Protein Side	[Drug-Zn] Drug Side
1	11.4 μM	9.1 μM
2	11.1 μM	9.3 μM
3	7.7 μM	8.2 μM
4	6.5 μM	6.1 μM

B

wt (20 μM)

Trial #	[Drug-Zn] Protein Side	[Drug-Zn] Drug Side
1	6.8 μM	5.2 μM
2	4.2 μM	3.4 μM
3	6.0 μM	5.0 μM
4	6.0 μM	4.8 μM

References

1. Butler JS and Loh SN. Structure, function, and aggregation of the zinc-free form of the p53 DNA binding domain. Biochemistry. 2003; 42(8):2396-2403.
2. Bullock AN, Henckel J, DeDecker BS, Johnson CM, Nikolova PV, Proctor MR, Lane DP and Fersht AR. Thermodynamic stability of wild-type and mutant p53 core domain. Proc Natl Acad Sci U S A. 1997; 94(26):14338-14342.
3. Diaz JF and Buey RM. Characterizing ligand-microtubule binding by competition methods. Methods Mol Med. 2007; 137:245-260.
4. Hunt JB, Neece SH and Ginsburg A. The use of 4-(2-pyridylazo)resorcinol in studies of zinc release from Escherichia coli aspartate transcarbamoylase. Anal Biochem. 1985; 146(1):150-157.
5. Butler JS and Loh SN. Zn(2+)-dependent misfolding of the p53 DNA binding domain. Biochemistry. 2007; 46(10):2630-2639.
6. Lokshin M, Li Y, Gaiddon C and Prives C. p53 and p73 display common and distinct requirements for sequence specific binding to DNA. Nucleic Acids Res. 2007; 35(1):340-352.
