# Supplementary material for: Small molecule restoration of wildtype structure and function of mutant p53 using a novel zinc-metallochaperone based mechanism
Source: Oncotarget. 2014 Sep 3;5(19):8879–92. doi: 10.18632/oncotarget.2432 (PMC4253404; doi:10.18632/oncotarget.2432)
Supplement: Supplementary file 2 [file oncotarget-05-8879-s002.pdf]

Table S1. Equilibrium dialysis reveals minimal binding between DBD and ZMC1. 20 μM ZMC1 + 10 μM $ZnCl_2$ was dialyzed against the indicated concentration of protein. Every unit of concentration represents 1/2 of a 726-Zn complex measured by absorbance at 370 nm. Higher concentrations were attempted but failed as the drug would stick to the equilibrium dialysis vessels.

A

r175h (19.3 μM)

Trial #	[Drug-Zn] Protein Side	[Drug-Zn] Drug Side
1	11.4 μM	9.1 μM
2	11.1 μM	9.3 μM
3	7.7 μM	8.2 μM
4	6.5 μM	6.1 μM

B

wt (20 μM)

Trial #	[Drug-Zn] Protein Side	[Drug-Zn] Drug Side
1	6.8 μM	5.2 μM
2	4.2 μM	3.4 μM
3	6.0 μM	5.0 μM
4	6.0 μM	4.8 μM
